# Three-dimensional kinematic evaluation of scapulohumeral rhythm after reverse shoulder arthroplasty: a systematic review and meta-analysis

**DOI:** 10.1016/j.xrrt.2021.10.009

**Published:** 2021-12-06

**Authors:** Felipe F. Gonzalez, Raphael Fonseca, Gustavo Leporace, Rafael Pitta, Marcos N. Giordano, Jorge Chahla, Leonardo Metsavaht

**Affiliations:** a Department of Orthopedic Surgery, Galeão Air Force Hospital (Hospital de Força Aérea do Galeão), Rio de Janeiro, Brazil; b Brazil Institute of Health Technologies (Instituto Brasil de Tecnologias da Saúde), Rio de Janeiro, Brazil; c Post Graduation Program of Clinical Radiology, Federal University of São Paulo (Universidade Federal de São Paulo), São Paulo, Brazil; d Midwest Orthopaedics at Rush University Medical Center, Chicago, IL, USA

**Keywords:** Scapulohumeral rhythm, 3D motion analysis, Reverse shoulder arthroplasty, Contact forces, Complication, Kinematic

## Abstract

**Background:**

The movement of the arm relative to the trunk results from 3-dimensional (3D) coordinated movements of the glenohumeral (GH) and scapulothoracic (ST) joints and dictates the scapulohumeral rhythm (SHR). Alterations in SHR increase joint overload and may lead to low functional scores, pain, and failures in patients undergoing reverse total shoulder arthroplasty (RSA). The goal of this systematic review and meta-analysis was to examine 3D SHR kinematics after RSA and compare it to that of asymptomatic shoulders.

**Methods:**

A systematic review and meta-analysis of articles in English were performed using PubMed, Embase, Cochrane Library, and SciELO. Additional studies were identified by searching bibliographies. Search terms included “Reverse shoulder arthroplasty”, “3D”, and “scapula”. It was selected cross-sectional studies that reported SHR with 3D motion analysis systems in patients who underwent RSA and asymptomatic controls. Two authors independently performed the extraction of articles using predefined data fields, including study quality indicators.

**Results:**

Data from four studies were included in quantitative analysis, totaling 48 shoulders with RSA and 63 asymptomatic shoulders. Pooled analyses were based on random-effects model (DerSimonian-Laird). A statistically smaller SHR ratio was observed in the RSA group than that in the control group (*P* < .00001), meaning a greater contribution of ST joint in relation to GH joint for arm elevation. The standardized mean difference was −1.16 (95% confidence interval: −1.64, −0.67). A sensitivity analysis with three more studies that had imputed data on control group did not change the direction of the effect. The standardized mean difference on sensitivity analysis was −0.60 (*P* = .03; 95% confidence interval: −1.13, −0.06). It was detected as “not important heterogeneity” within the comparison (I^2^: 22%). Chi-square was not statistically significant (Chi^2^: 3.85), and I^2^ was 22%. Tau^2^ was not zero (Tau^2^: 0.05). Sensitivity analysis showed an I^2^ of 74%, which might represent substantial heterogeneity, Chi-square was not statistically significant (Chi^2^: 23.01), and Tau^2^ was not zero (Tau^2^: 0.37).

**Conclusion:**

This study found that RSA shoulders have an increased contribution of ST joint during arm elevation, compared with asymptomatic shoulders. More movement in ST joint in proportion to GH joint increases GH joint contact forces, which could lead to component loosening or other complications. Further studies should address the clinical implications of this kinematic finding.

Since its conception, reverse total shoulder arthroplasty (RSA) has gained significant popularity, especially due to the substantial clinical and functional improvement in patients with rotator cuff arthropathy.^44^ Designed in 1993, it was developed as an alternative to anatomic shoulder replacement in patients with massive rotator cuff tears.^35^ Recently, RSA indications have been expanded to acute three- or four-part proximal humerus fractures in the elderly, primary osteoarthritis, chronic anterior dislocation, and failed anatomic total shoulder arthroplasty.^41^ As a consequence of its worldwide use, there has been an increase in the number of complications and reoperations.^36^

Multiple RSA complications are described in the literature. Complications on the glenoid side, humeral side, implant issues, and muscular complications, account for more than twenty different types of complications reported.^35^ Often, they are misunderstood and misdiagnosed, and this can lead to many reoperations on the same patient.^5^ Moreover, instability or dislocation of implants is frequently attributed to an imbalance of the muscles around the reconstructed joint^5^; however, those complications might be attributed to kinematic differences between the anatomic and prosthetic joints. A comprehensive understanding of RSA biomechanics is crucial to identify how complications can be avoided and/or treated in the event they are already present.

Recent studies have focused on the contribution of the scapulothoracic (ST) joint motion to total humerothoracic (HT) motion or arm motion, which is composed of ST and glenohumeral (GH) joint motion.^20^^,^^21^^,^^34^^,^^43^ Scapulohumeral rhythm (SHR) has been used to quantify the relative motion of the scapula and humerus to total shoulder motion and is defined by GH elevation divided by ST superior rotation motion (Fig. 1).^2^^,^^7^^,^^19^^,^^34^ SHR has been widely used as a sensitive measure of shoulder dysfunction and can provide important information regarding current or future shoulder injuries.^3^^,^^24^^,^^27^^,^^42^

**Figure 1:**
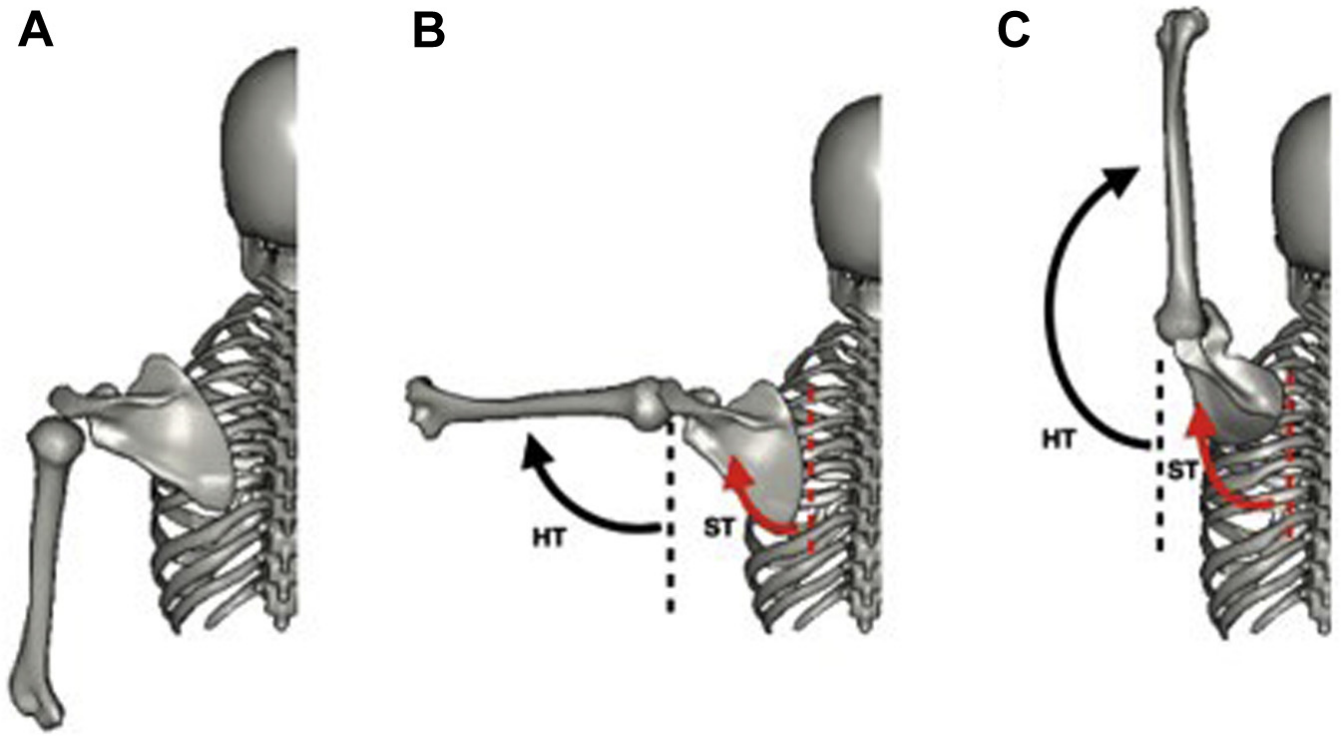
Scapulohumeral rhythm (SHR) during shoulder abduction. Note: SHR is the relation between the movement of GH and ST joints during arm elevation and the movement of the humerus relative to the trunk. *Dorsal view* of left shoulder girdle. (**A**) Shoulder abduction at 0°; (**B**) shoulder abduction at 90°; (**C**) maximum shoulder abduction. **: humerothoracic (HT) elevation; **: scapulothoracic (ST) superior rotation.

Many of the studies analyzing the HT motion after RSA use 2D methods, 3D computed tomographic models, or cadaveric biomechanical tests to quantify the range of motion.^6^^,^^8^^,^^18^^,^^31^^,^^33^^,^^43^ Two-dimensional methods to quantify GH joint motion are limited and may result in inaccurate or incomplete descriptions of joint position. Moreover, 3-dimensional (3D) computed tomographic models or cadaveric biomechanical results may not be extrapolated to an *in vivo* setting. On the other hand, 3D skin-based motion systems have shown to be accurate and reliable for scapular and humeral kinematics in vivo.^14^^,^^16^^,^^22^^,^^29^ Although studies that used 3D motion analysis to measure shoulder function after RSA show altered SHR, not all of them reported SHR as the primary outcome or compared it to a control group.^1^^,^^2^^,^^4^^,^^7^ To the best of our knowledge, there is no systematic review that addresses these methodological issues. Therefore, an accurate understanding of 3D SHR on patients submitted to RSA is still lacking. The goal of this systematic review and meta-analysis was to overcome these methodological limitations and examine 3D SHR kinematics after RSA and compare it to that of asymptomatic shoulders.

## Materials and methods

### Eligibility criteria

#### Types of studies

Studies evaluating SHR in the scapular abduction plane with 3D motion analysis systems in patients who underwent RSA and in asymptomatic shoulders were included. No design, publication date, and publication status restrictions were imposed. The search was restricted to studies in English language.

#### Types of participants

Participants of any age, any gender, any race, any socioeconomic status, any functional level, at any time after surgery, and with any indication for RSA were included in this analysis. There were no exclusion criteria based on participants' characteristics.

#### Types of exposure

This review was limited to studies looking for SHR in patients with RSA.

#### Types of outcome measure

The primary outcome measure is a performance-based outcome, the SHR.

### Information sources

Studies were primarily identified by research in electronic databases. Secondary identification was by expert consultation and scanning reference lists in similar articles. This search was applied to PubMed, Embase, Cochrane Library, and SciELO in January 17, 2020, at 2 pm.

### Search strategy

We used the following search terms combined in each search database with technical adaptations of Boolean operators for each database: ((Shoulder Replacement∗) OR (Shoulder arthroplast∗) OR (Shoulder Prosthesis)) AND ((Range of Motion) OR (Range of Movement) OR Flexion OR Extension OR Abduction OR Adduction OR Rotation OR Sagittal OR Coronal OR Axial OR Biomechanics OR Kinematic∗ OR Scapul∗)) AND ((3-dimensional OR 3D OR three-dimensional)).

### Study selection

Eligibility assessment was made by two investigators following the reporting guidelines for meta-analysis Preferred Reporting Items for Systematic Reviews and Meta-Analyses (PRISMA)^23^ and Meta-analyses of Observational Studies in Epidemiology (MOOSE).^38^

### Data collection process

We have developed a data extraction sheet containing relevant information. The extraction sheet was refined after the initial round. A total of 3 rounds of research looking for tables, graphs, and text were carried out by 2 investigators.

### Risk of bias in individual studies

We assessed each study quality using National Institutes of Health Quality Assessment Tool for Observational Cohort and Cross-Sectional Studies.^39^ Two investigators, in consensus, rated each study after full-text analysis. No clinical trial was included in this analysis because of eligibility criteria.

### Summary measures

The standard mean difference in SHR ratio comparing RSA and control group was the primary measure of treatment effect, using a random-effects model. Quantitative analyses were performed with all included studies. A standard mean difference was calculated instead of mean difference because different equipment and methodologies were used between studies. *P* value and 95% confidence intervals were calculated. *P* value was considered statistically significant when ≤.05.

### Planned methods of analysis

In two studies, SHR was reported in different parts of the total arm elevation.^19^^,^^34^ Mean and standard deviation (SD) were calculated for total arm elevation using the following formula: $\frac{Dz}{z}=\sqrt{{\left(\frac{Da}{a}\right)}^{2}+{\left(\frac{Db}{b}\right)}^{2}+{\left(\frac{Dc}{c}\right)}^{2}}$, divided by appropriate constants. Consider mean as “a”, “b”, or “c” and its associated SD or error as Da, Db, or Dc, respectively.^15^

One study reported range instead of SD.^21^ SD was estimated using the following formula: SD = (maximum value − minimum value)/4.^15^

One study did not report SHR of comparison group on text.^7^ We retrieved it from one figure. The same study did not report SDs of RSA and comparison group. We retrieved it from calculations following Cochrane Handbook for Systematic Reviews of Interventions^15^ recommendations using available data.

Heterogeneity was tested with Chi^2^ test and I^2^ statistics. Variance estimation among the effects observed in different studies (between-study variance) was calculated with Tau^2^.

The interpretation of heterogeneity followed Cochrane Handbook for Systematic Reviews of Interventions.^15^ Chi^2^ test was interpreted as statistically significant if <0.05. I^2^ statistics followed this guide for interpretation: 0% to 40%, might not be important; 30% to 60%, may represent moderate heterogeneity; 50% to 90%, may represent substantial heterogeneity; 75% to 100%, considerable heterogeneity.

Statistical analysis was performed with Review Manager software, version 5.4.

### Sensitivity analysis

A sensitivity analysis was conducted to assess differences in the overall effect adding studies that had imputed data. Other analyses such as subgroup or meta-regression were not performed.

Among the studies included in the sensitivity analysis, one study did not report SHR, but GH elevation and ST upward rotation.^1^ The SHR value was obtained by dividing GH by ST. Means and SDs were calculated using the following formulas: $z\pm Dz=\frac{a\pm Da\cdot b\pm Db}{c\pm Dc}$ and $\frac{Dz}{z}=\sqrt{{\left(\frac{Da}{a}\right)}^{2}+{\left(\frac{Db}{b}\right)}^{2}+{\left(\frac{Dc}{c}\right)}^{2}}$. Consider mean as “a”, “b”, or “c” and its associated SD or error as Da, Db, or Dc, respectively.^15^

Two studies included in sensitivity analysis did not have asymptomatic shoulders as the control group,^1^^,^^2^ and one study did not have the control group.^4^ Means and SDs were imputed as “worst-case scenario” based on summary measures reported on other studies included in the quantitative analysis.^19^^,^^21^^,^^32^

### Risk of bias across studies

Risk of bias across studies was assessed by a funnel plot in a way of displaying potential publication bias.

## Results

### Study selection

**Figure 2:**
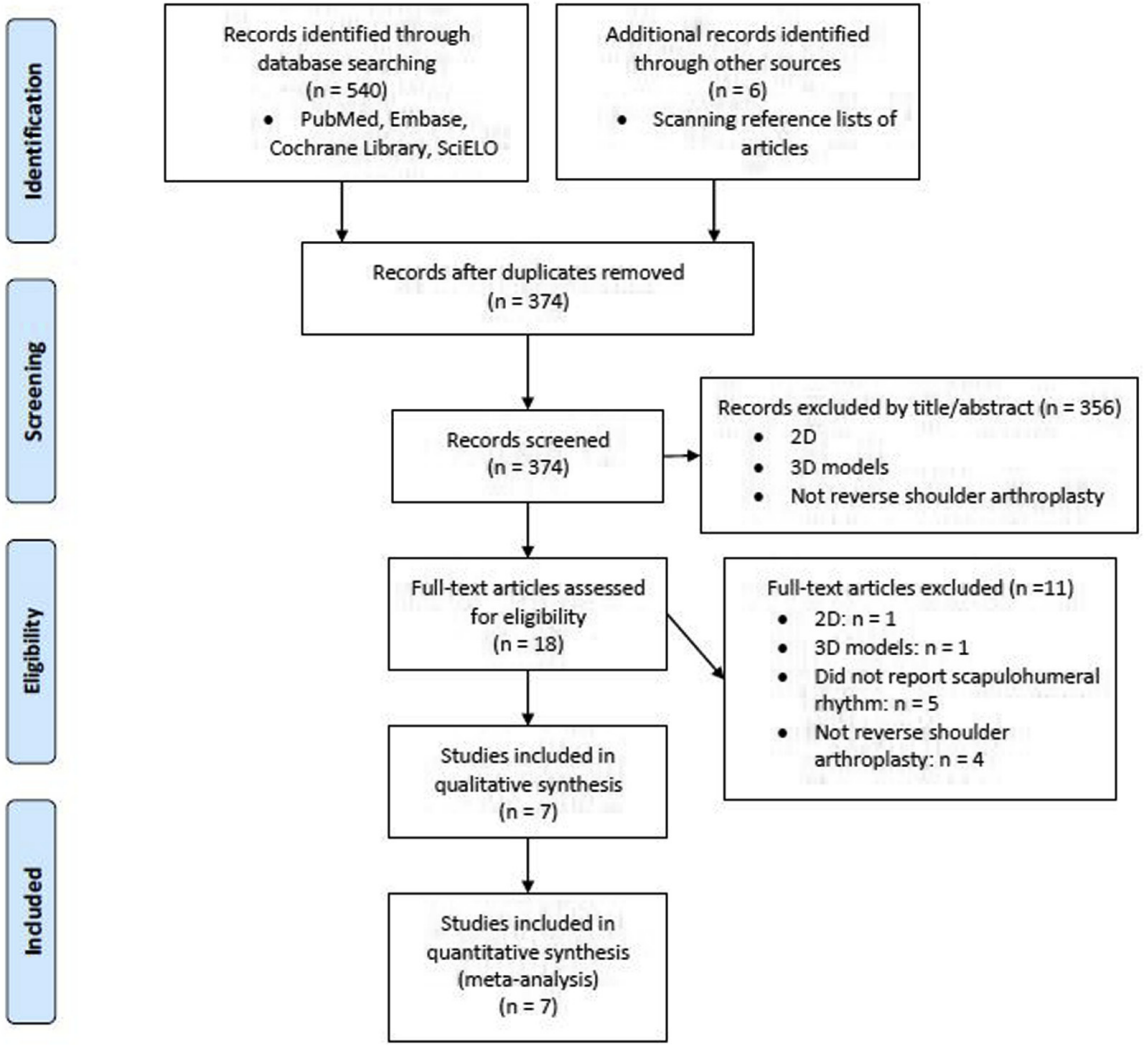
PRISMA 2009 (Preferred Reporting Items for Systematic Reviews and Meta-Analyses) flow diagram of the study.

We have identified 540 records through database searching (PubMed, Embase, Cochrane Library, and SciELO). Additionally 6 articles were identified through reference lists of identified articles. After the removal of duplicates, 374 articles had their titles and/or abstracts screened. Owing to a clear misfit of eligibility criteria, 356 articles were excluded. Eighteen articles were assessed for full-text screening in more detail, and 11 were excluded because of the use of a 2D analysis system, 3D models, or for not reporting SHR or any motion pattern that could enable SHR calculation (eg, GH and ST range of motion) (Fig. 2). No unpublished relevant studies were obtained.

### Study characteristics

The following subsections explain the overall characteristics of the included studies.

#### Methods

All seven studies finally selected for the review had a cross-sectional design and were published in English. Six studies used electromagnetic 3D systems for motion analysis,^1^^,^^2^^,^^4^^,^^7^^,^^19^^,^^32^ and one used an optical 3D motion analysis system.^21^ All analyzes were conducted in a laboratory.

#### Participants

A total of 48 shoulders with RSA and 63 asymptomatic shoulders were analyzed. The sensitivity analysis included a total of 117 shoulders with RSA, and 63 asymptomatic shoulders were analyzed. Participants were tested at a range of 2 to 96 months after surgery. The mean age of the patients with RSA was 71.80 years (range: 53-85) and controls 61.38 (range: 22-79).

#### Exposure

The exposure being analyzed was RSA. One study included RSA with lateralized center of rotation.^19^ Two studies included revision RSA among primary RSA group.^1^^,^^7^

#### Outcome

In all studies, the primary outcome assessed was SHR during active arm elevation in scapular plane. Only one study measured abduction on frontal plane,^34^ instead of abduction on scapular plane. Scapular plane was defined in studies as 30° to 45° from the frontal plane. All of them were without weight-bearing and with active movements. Three studies used maximal active joint angles (peak) to calculate SHR,^1^^,^^2^^,^^4^ and others used mean SHR during arm elevation. In three studies, SHR was retrieved from calculation using GH and ST angles.^1^^,^^19^^,^^34^

#### Control

Six studies reported the use of control groups.^1^^,^^2^^,^^7^^,^^19^^,^^21^^,^^34^ Three reported asymptomatic individuals,^7^^,^^19^^,^^34^ and one reported asymptomatic contralateral shoulder as control.^21^ One study reported total shoulder anatomic arthroplasties as the control group,^2^ and one reported revision RSA as the control group^1^; therefore, their control group data were not used in the analysis.

### Risk of bias in individual studies

We assessed overall quality and risk of bias using Quality Assessment Tool for Observational Cohort and Cross-Sectional Studies.^30^ According to this tool, all studies had moderate risk of bias and moderate quality (Table I).

**Table I:** NIH quality assessment tool for observational cohort and cross-sectional studies.

Author/year of publication							
Criteria	∗Bergmann 2008^4^	∗Alta 2011^1^	De Toledo 2012^7^	Kwon 2012^19^	∗Alta 2014^2^	Lee 2016^21^	Roren 2017^34^
1. Was the research question or objective in this paper clearly stated?	Yes	Yes	Yes	Yes	Yes	Yes	Yes
2. Was the study population clearly specified and defined?	No	Yes	No	No	Yes	No	Yes
3. Was the participation rate of eligible persons at least 50%?	NR	NR	NR	NR	NR	NR	NR
4. Were all the subjects selected or recruited from the same or similar populations (including the same time period)? Were inclusion and exclusion criteria for being in the study prespecified and applied uniformly to all participants?	No	Yes	No	No	No	No	Yes
5. Was a sample size justification, power description, or variance and effect estimates provided?	No	No	No	No	No	No	No
6. For the analyses in this paper, were the exposure(s) of interest measured prior to the outcome(s) being measured?	NA	NA	NA	NA	NA	NA	NA
7. Was the timeframe sufficient so that one could reasonably expect to see an association between exposure and outcome if it existed?	Yes	Yes	Yes	Yes	Yes	Yes	Yes
8. For exposures that can vary in amount or level, did the study examine different levels of the exposure as related to the outcome (e.g., categories of exposure, or exposure measured as continuous variable)?	NR	NR	NR	NR	NR	NR	NR
9. Were the exposure measures (independent variables) clearly defined, valid, reliable, and implemented consistently across all study participants?	Yes	Yes	Yes	Yes	Yes	Yes	Yes
10. Was the exposure(s) assessed more than once over time?	NR	NR	NR	NR	NR	NR	NR
11. Were the outcome measures (dependent variables) clearly defined, valid, reliable, and implemented consistently across all study participants?	Yes	Yes	Yes	Yes	Yes	Yes	Yes
12. Were the outcome assessors blinded to the exposure status of participants?	No	No	No	No	No	No	No
13. Was loss to follow-up after baseline 20% or less?	Yes	Yes	Yes	Yes	Yes	Yes	Yes
14. Were key potential confounding variables measured and adjusted statistically for their impact on the relationship between exposure(s) and outcome(s)?	No	No	No	No	No	No	No
15. Quality rating (good, fair, poor)	Fair	Good	Fair	Fair	Fait	Fair	Good

*NR*, not reported; *NA*, not applicable.

∗ Studies included in sensitivity analysis.

### Results from individual studies

See Table II for summary outcomes of individual studies including SHR, SDs, and sample sizes from RSA and asymptomatic groups.

**Table II:** Summary characteristics of individual studies: outcomes.

Name	Year of publication	RSA group (n)	SHR or GH:ST (mean)	SD	Control group (n)	SHR or GH:ST (mean)	SD
De Toledo	2012	9	1.10:1	0.43	15	1.50:1	0.43
Kwon	2012	17	2.23:1	1.02	12	4.76:1	3.34
Lee	2016	13	1.27:1	0.12	13	1.92:1	0.43
Roren	2017	9	1.10:1	0.60	23	2.20:1	1.44
Studies included in sensitivity analysis							
Begmann	2008	18	1.60:1	0.40	**18**	**1.50:1**	**3.34**
Alta	2011	35	1.75:1	1.05	**35**	**1.50:1**	**3.34**
Alta	2014	16	1.49:1	0.30	**16**	**1.50:1**	**3.34**

*RSA*, reverse shoulder arthroplasty; *N*, number of shoulders; *SHR*, scapulohumeral rhythm; *SD*, standard deviation; *GH*, glenohumeral; *ST*, scapulothoracic.

Values in bold represent imputed data.

See Table III for summary characteristics of individual studies including age of participants, time after surgery, angle analyzed during arm elevation, and type of equipment used for 3D motion analysis.

**Table III:** Summary characteristics of individual studies.

Name (Year of publication)	Control group	Age RSA (range)	Age control (range)	Time after surgery	Angle analyzed	Measure
De Toledo (2012)^7^	Asymptomatic	73.3 (SD: 9.4)	25.1 (SD: 3.7)	Mean: 67 mo	15°, 30°, 45°, 60°, 75°, and 90°	Electromagnetic
Kwon (2012)^19^	Asymptomatic	69.8 (SD: 5.8)	68.2 (SD: 11.4)	>6 mo	30°, 50°, 70°, 90°	Electromagnetic
Lee (2016)^21^	Contralateral	72 (69-79)	72 (69-79)	13-48 mo	Each 10° of arm elevation	Optical
Roren (2017)^34^	Asymptomatic	78.7 (SD: 6.9)	75.5 (SD: 7.6)	3-96 mo	30°, 60°, 90°	Electromagnetic
Studies included in sensitivity analysis						
Bergmann (2008)^4^	No control	71 (58-85)	-	2-68 mo	Peak	Electromagnetic
Alta (2011)^1^	Revision	71 (58-85)	-	4-63 mo	Peak	Electromagnetic
Alta (2014)^2^	TSA	72 (53-87)	-	6-41 mo	Peak	Electromagnetic

*Revision*, revision prosthesis; *RSA*, reverse shoulder arthroplasty; *TSA*, anatomic total shoulder arthroplasty.

Age is presented in years (range) or standard deviation (SD).

### Syntheses of results

**Figure 3:**
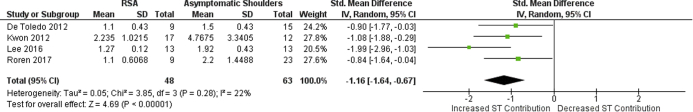
Scapulohumeral rhythm ratio standardized mean differences between RSA and asymptomatic shoulders. Note: The diamond on “increased ST contribution” indicates that SHR is smaller in the RSA group (greater contribution of scapulothoracic joint in relation to glenohumeral joint for arm elevation).

Refer to Fig. 3 for SHR ratio standard mean differences between RSA and asymptomatic shoulders.

SHR data were available for all studies included in the analysis, totaling 48 shoulders with RSA and 63 asymptomatic shoulders. A statistically smaller SHR was observed in the RSA group than that in the control group (*P* < .00001), meaning a greater contribution of ST joint in proportion to GH joint for arm elevation. Standardized mean difference was −1.16 (95% CI: −1.64, −0.67).

### Sensitivity analysis

**Figure 4:**
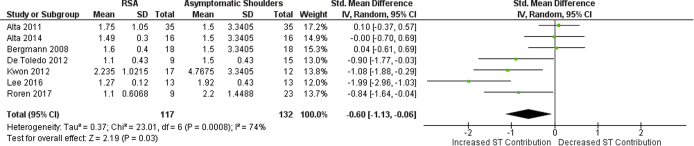
Sensitivity analysis: scapulohumeral rhythm ratio standardized mean differences between RSA and asymptomatic shoulders. Note: The diamond on “increased ST contribution” indicates that SHR is smaller in the RSA group (greater contribution of scapulothoracic joint in relation to glenohumeral joint for arm elevation).

A sensitivity analysis was conducted to assess differences in the overall effect by adding studies that had imputed data on SHR ratio and SDs of control group (Fig. 4). A total of 117 shoulders with RSA and 63 asymptomatic shoulders were analyzed. It was observed that adding three studies with imputed data did not change the direction of the difference between groups. A statistically smaller SHR ratio was detected in the RSA group than that in the control group (*P* = .03), also meaning a greater contribution of ST in proportion to GH joint for arm elevation. Standardized mean difference was −0.60 (95% CI: −1.13, −0.06). Other analyses such as subgroup or meta-regression were not performed.

### Risk of bias across studies

It was detected not important heterogeneity within the comparison (I^2^: 22%). Chi-square was not statistically significant (Chi^2^: 3.85), and I^2^ was 22%. Tau^2^ was not zero (Tau^2^: 0.05).

Sensitivity analysis showed an I^2^ of 74%, which might represent substantial heterogeneity. Chi-square was not statistically significant (Chi^2^: 23.01), and Tau^2^ was not zero (Tau^2^: 0.37).

**Figure 5:**
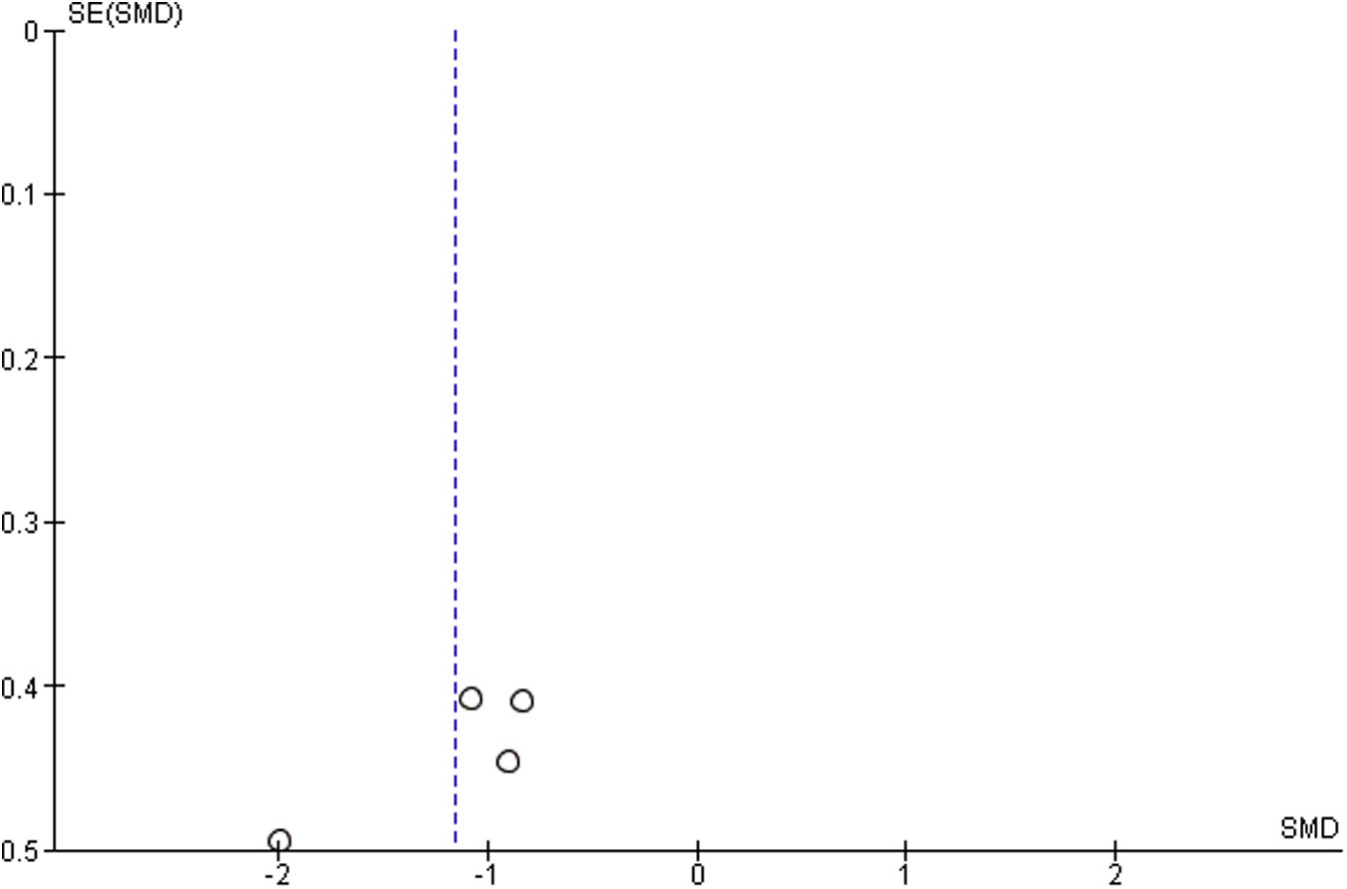
Funnel plot. Note: asymmetric pattern, with no study demonstrating positive difference of mean and all studies having a similar sample size.

Funnel plot including studies without imputed data showed an asymmetric pattern, with no study demonstrating positive difference of mean and all studies having a similar sample size (Fig. 5). There is no clear evidence of publication bias. However, this analysis is limited because of having only four studies.

**Figure 6:**
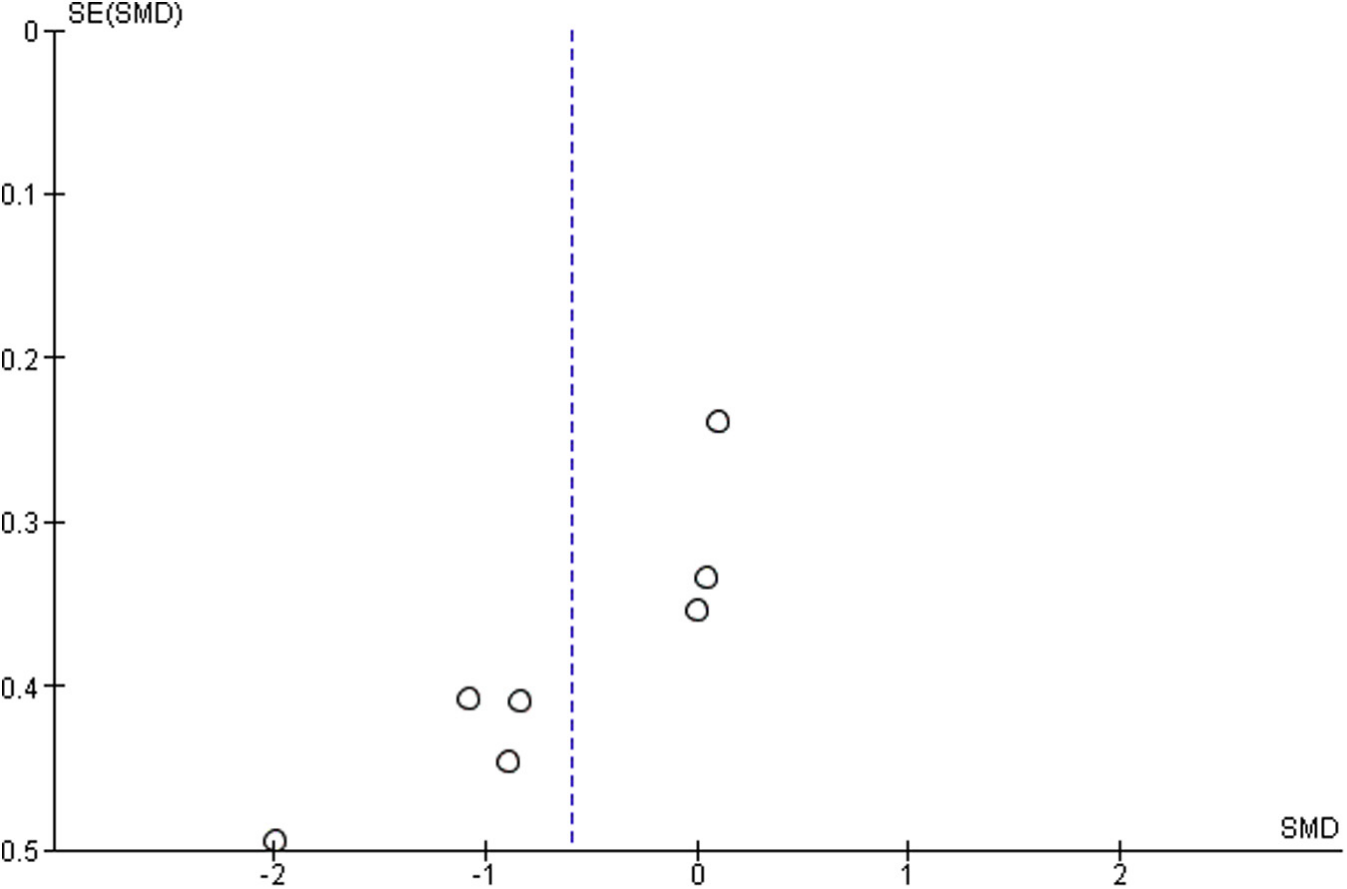
Funnel plot: sensitivity analysis. Note: slightly asymmetric pattern, with studies demonstrating negative or no difference of mean and no study demonstrating positive difference of mean. Sample size is also similar.

The funnel plot of the studies included in sensitivity analysis showed a slightly asymmetric pattern, with studies demonstrating negative or no difference of mean and no study demonstrating positive difference of mean (Fig. 6). There is no clear evidence of publication bias. However, this analysis is limited because of the inclusion of only seven studies.

## Discussion

The main result of this study evaluating 3D SHR kinematics of shoulders was an increased ST motion relative to GH motion in patients who underwent RSA with scapular plane abduction. Such finding demonstrates a decreased SHR when compared with asymptomatic shoulders. None of the studies included in the analysis showed increased SHR for the RSA group.

The present study helps to understand RSA biomechanics in individuals who underwent surgery by different indications, conditions, and at different points of time after surgery. It was possible to analyze patients who had humeral head fractures, rotator cuff disease, massive rotator cuff tear, and arthropathies and had undergone primary RSA and revision RSA, in a wide range of time points after surgery (2-96 months), demonstrating an important clinical heterogeneity in this population. Motion contributions to arm elevation may vary widely over these time points, but also, there is contrary evidence showing that shoulder kinematics after RSA do not show significant differences after six months.^25^ Studies included in this analysis report an assessment at least six months after surgery, probably reducing some heterogeneity. Finally, this wide time frame and the different indications and conditions of RSA possibly improve the external validity of the study.

For a more useful analysis of the effects of the prosthesis in shoulder kinematics, more studies have measured the two separate parts of the HT motion, the GH joint motion and the ST joint motion, independently. SHR has been used as a measure of shoulder dysfunction in many studies.^9^^,^^17^^,^^32^^,^^43^ In asymptomatic shoulders, SHR ratio has been reported to range from 1.35:1 to 7.9:1, whereas in patients after RSA, from 1.3:1 to 2.4:1.^19^^,^^43^ Our results showed similar ranges of SHR ratio in asymptomatic shoulders (1.5:1 to 4.76:1) and RSA (1.10:1 to 2.23:1).

Endorsing as with our observation, recent studies investigating shoulders with massive rotator cuff tears and different grades of arthropathy have found a greater contribution of ST joints between 30° and 90° of elevation on scapular plane, when compared with asymptomatic shoulders.^46^^,^^48^ Furthermore, pseudoparalytic patients may have none or even negative contribution of the GH motion due to gravity.^46^ These reports suggest that patients who underwent RSA due to rotator cuff arthropathy may present different biomechanics compared with normal shoulders, with increased utilization of ST joint already before surgery.

Clinically, there is a concern about declining implant survivorship over time and high complication rates in RSA.^13^ Moreover, if the complication cause is undetected, leading to reoperation, functional outcome may be affected.^5^ A recent study has found the SHR affects GH joint force.^10^ The authors found that increasing the ratio of ST to GH elevation increased GH joint forces. Increased ST motion to arm elevation and GH joint forces may overstress periscapular structures or even the RSA implant, leading to component loosening or other complications.

Most of the studies analyzed only reported upward rotation in RSA, a scapular motion addressed at SHR that occurs in the coronal plane.^2^^,^^7^^,^^19^^,^^34^ Future studies should look at the other planes of motion of ST joint, not only upward rotation. The 3D scapular motion includes internal/external rotation, posterior/anterior tilt, and upward/downward rotation. Those studies may be insufficient to accurately describe the complex scapular kinematics, as other impairments could possibly occur in sagittal or transverse planes.^2^^,^^7^^,^^19^^,^^34^ However, Lee et al,^21^ analyzing the elevation along with sagittal plane arm flexion and scapular plane arm abduction, found that RSA affected only upward rotation of the 3D scapular motion and that internal rotation and posterior tilting of the scapula were not significantly different when compared with contralateral shoulders.^21^ Therefore, studies that used only upward rotation of the scapula, without looking at other planes of motion, may be overlooking important features of scapular kinematics for RSA. However, we have to consider sagittal and transverse plane scapular movements have increased coefficient of variation, demanding a greater sample size to achieve significant statistical power to reduce bias.^12^

Because of several factors, the present study encounters limitations. Our data rely on those reported in the included studies, and we are, therefore, limited by the clarity in which those results are reported, as well as by the study design and level of evidence of the included works. For this reason, we used the National Institutes of Health Quality Assessment Tool for Observational Cohort and Cross-Sectional Studies to evaluate the quality of the included studies, which overall were found to have fair quality (Table I). Also, we looked for publication bias on two funnel plots (Figure 5, Figure 6), finding no suggestion of publication bias but a limited quantity of total researches. A limitation of our analysis of the functional outcomes is that some comparative studies did not report SDs or asymptomatic shoulders as control groups. Cochrane Handbook for Systematic Reviews of Interventions^15^ states as a general rule that the missing summary data should not be a reason for excluding a study, and this assertion is an agreement to PRISMA Statement^23^ and other authors.^11^^,^^45^ On one hand, valuable information would be lost if those studies were excluded, and on the other hand, a high degree of uncertainty would be added to the analysis if they were included. We decided for including those studies and imputing the “worst” values reported in studies included in this analysis as the “worst-case scenario” method is a conservative approach. We have found in a sensitivity analysis that the inclusion of those studies with imputed data did not affect the overall effect of reduction of SHR, which gives more robustness of findings.

Furthermore, our data showed a moderate level of heterogeneity and may be subject to treatment-bias effect. Specifically, 3D motion analyses were different between the studies, which may have an impact on our findings because of differences in instrumentation, planes of analysis, definition of axis orientation, determination of angular value around the starting position, measuring range, trunk position, types of subjects, and the use of static versus dynamic motion.^26^ Also, one study^19^ included lateralized RSA, where scapular biomechanics are not fully understood yet and may differ from nonlateralized RSA. Although, to our best knowledge, there is no evidence that SHR differs between those implants, there are studies that show that lateralized RSAs have greater arm external rotation and lesser scapular notching.^12^^,^^28^^,^^37^ To limit bias introduced by heterogeneity, a random-effects model was used to perform the meta-analyses (DerSimonian-Laird). To address measurement differences, standard mean differences were used instead of mean differences. Although a potential drawback, the heterogeneous data are reflective of the reality of clinical practice and may, therefore, most accurately represent what should, actually, be encountered in the clinical setting.

Another limitation is the lack of ideal control data from an age-matched control group without rotator cuff pathology. A noninjured control group is hard to obtain because of the prevalence rates of asymptomatic cuff tears of 31% in individuals aged between 70 and 79 years and 51% in individuals older than 80 years.^40^ A possible alternative is the use of the contralateral shoulder in the same patient, but Yamaguchi et al indicated a 50% likelihood of a bilateral tear after the age of 66 years.^47^ Opportunely, the control group had a mean age less than 66 years (61.38 years) and 10 years less than that of the RSA group (71.80 years; range: 53—85), with a wider range (22-79 years), possibly reducing the prevalence of subjects with asymptomatic cuff tears. A wider range, on one hand, also increases the generalizability of the results of the study, but on the other hand, it can introduce bias because young and old subjects could have different kinematics.

## Conclusion

This study found a major contribution of the scapular motion in proportion to GH motion after RSA on the scapular abduction plane, leading to a decrease in SHR when compared with asymptomatic shoulders. More movement in ST joint in proportion to GH joint increases GH joint contact forces, which could lead to component loosening or other complications. Future studies should address how it can impact preoperative patient selection, surgery decision-making, and postoperative care.

## Disclaimer

Funding: No funding was disclosed by the authors.

Conflicts of interest: The authors, their immediate families, and any research foundation with which they are affiliated have not received any financial payments or other benefits from any commercial entity related to the subject of this article.
